# Evaluation of the effects of the hydroalcoholic extract of *Terminalia chebula* fruits on diazinon-induced liver toxicity and oxidative stress in rats

**Published:** 2017

**Authors:** Raheleh Ahmadi-Naji, Esfandiar Heidarian, Keihan Ghatreh-Samani

**Affiliations:** 1 *Student Research Committee, Shahrekord University of Medical Sciences, Shahrekord, Iran *; 2 *Clinical Biochemistry Research Center, Basic Health Sciences Institute, Shahrekord University of Medical Sciences, Shahrekord, Iran *; 3 *Medical Plants Research Center, Basic Health Sciences Institute, Shahrekord University of Medical Sciences, Shahrekord, Iran*

**Keywords:** Terminalia chebula, Diazinon, Hepatotoxicity, Oxidative stress, TNF-α, Liver damage

## Abstract

**Objective:**

Diazinon causes oxidative stress and dysfunction of the liver. This study was undertaken to evaluate the effects of the hydroalcoholic extract of *Terminalia chebula*, on some biochemical and histopathological parameters of liver tissue in diazinon-administered rats.

**Materials and Methods:**

Wistar rats were orally administered with 25 mg/kg body weight diazinon. Vehicle (distilled water) and silymarin (50 mg/kg body weight) were used as the negative and positive control groups, respectively. Diazinon-administered groups were treated with *T.* *chebula* (*Terminalia chebula*) fruit extract (200, 400, and 800 mg/kg). After 15 days of treatment, the blood specimens and liver samples were examined.

**Results:**

In diazinon-treated group, the levels of serum urea, high density lipoprotein (HDL), and liver superoxide dismutase (SOD), catalase (CAT), and vitamin C significantly decreased (p<0.05) compared to control. Also, in this group, serum triglyceride (TG), total cholesterol (TC), very low density lipoprotein cholesterol (VLDL), protein carbonyl (PC), malondialdehyde, tumor necrosis factor-α (TNF-α), and *TNF-α* gene expression significantly increased (p<0.05) as compared to the control (vehicle-treated rats). Treatment with *T. chebula* resulted in a significant increase (p<0.05) in CAT, SOD, vitamin C, HDL and a significant decrease (p<0.05) in the level of urea, MDA, PC, TG, TC, VLDL, TNF-α protein, and the gene expression of *TNF-α* compared with test without treatment group. Histopathological evidence demonstrated that treatment with *T. chebula* extract could decrease liver lymphocyte infiltration.

**Conclusion:**

The present study suggests that *T. chebula* fruit extract has protective effects against diazinon-induced oxidative stress.

## Introduction

Diazinon (O, O-diethyl-O-[2-isopropyl-6-methyl-4-pyrimidinyl] phosphorothioate) is one of the most commonly used organophosphate insecticides worldwide for different agricultural and gardening uses (El-Shenawy et al., 2010 ▶). The contamination of food and water with diazinon may increase humans exposure to it and leads to dysfunction of the liver, kidney, neurological system, and pancreas (El-Shenawy et al., 2010 ▶; Gokcimen et al., 2007 ▶). Some studies have demonstrated that diazinon-induced toxic effects lead to production of free radicals, depletion of antioxidants, and ultimately, induction of oxidative stress and cell damage (Ahmed et al., 2013 ▶). 

Medicinal plants greatly contribute to improvement of human body's antioxidant status (Lee et al., 2005 ▶). Nowadays, medicinal plants are attracting attention due to their inexpensiveness, safety, and fewer side effects (Chandra, 2012 ▶). Also, consumption of plant-derived compounds and fruits can reduce the risk of incidence of different diseases (Heidarian et al., 2011 ▶; Heidarian et al., 2016 ▶; Rezaei and Heidarian, 2013 ▶). The protective effects of some herbal agents have been already demonstrated on diazinon-induced toxicity in rats (Abdel-Daim, 2016 ▶; Al-Attar, 2015 ▶; Hassouna et al., 2015 ▶). 

*Terminalia chebula* is one of the medicinal plants that is found in India, Egypt, Turkey, and Pakistan. *T. chebula* has demonstrated antioxidant, antimicrobial, detoxifying, anti-diabetes, anti-ulcer, anticancer, antiviral, and antifungal effects (Chandra, 2012 ▶). In addition, beneficial effects of *T. chebula* have been confirmed on liver and gastric diseases (Bhattacharya et al., 2007 ▶; Lee et al., 2005 ▶). Therefore, the aim of this study was to investigate *T. chebula* effects on serum lipids, urea, ferric reducing antioxidant power (FRAP), protein carbonyl (PC), liver and serum malondialdehyde (MDA), serum tumor necrosis factor-α (TNF-α) and its gene expression, liver catalase (CAT), superoxide dismutase (SOD), vitamin C, and histopathology of liver tissues in diazinon-administered rats.

## Materials and Methods

Diazinon was purchased from Egrochimi Co. (Hungary, 60% purity). The kits of triglyceride (TG), total cholesterol (TC), glutamate oxaloacetate transaminase (GOT), glutamate pyruvate transaminase (GPT), low-density lipoprotein cholesterol (LDL-C), high density lipoprotein cholesterol (HDL-C), urea, and creatinine were purchased from Pars Azmoon Co. (Iran, Tehran). Nitro blue tetrazolium chloride (NBT), silymarin, and 6-tripyridyl-s-triazine (TPTZ) were provided from Sigma-Aldrich (St. Louis, MO, USA). SYBR^®^ Green PCR Master Mix was obtained from Qiagen Co. (Düsseldorf, Germany). 2-Thiobarbituric acid, coomassie brilliant blue G250, and hydrogen peroxide were purchased from Merck Co. (Darmstadt, Germany). All other chemicals used were of analytical grade.

**Plant material and extraction**

*T. chebula* was purchased from Medical Plants Research Center of Isfahan University of Medical Sciences, Isfahan, Iran. Also, a voucher specimen was deposited (herbarium no. 502). The fruits of *T. chebula* were air-dried at room temperature, then, it was grounded and ethanol:water (70:30, v/v) was used to prepare the extract. Finally, the obtained extract was kept at 5°C for further use (Farahpour et al., 2015 ▶). 

**Measuring antioxidant, flavonoid, and phenolic contents**

The flavonoid and phenolic contents of the hydroalcoholic extract of *T. chebula* fruits extract were measured according to the protocol used by Chang et al. (2002) ▶ and McDonald et al. (2001) ▶, respectively. Phenolic and flavonoid contents were expressed in mg/g gallic acid. Antioxidant activity was measured by DPPH according to Singh et al. (2008) ▶ study. Briefly, antioxidant activity was defined as the total antioxidant required to reduce the initial DPPH radical concentration to 50%.

**Animals and experimental design**

Forty eight male Wistar rats, weighing 200±20 g, were used in this study. All animals were kept under standard conditions (23±2°C temperature, 12hr-12hr light-dark cycle, and relative humidity of 55 ± 5%) and had free access to food and water, *ad libitum*. The rats were randomly assigned to six groups of eight each. Group 1 was the negative control (vehicle-treated group) which was orally treated with distilled water to exclude gavage-induced shock in other groups; Group 2 (diazinon-only group) received diazinon-only orally (25 mg/kg body weight, dissolved in distilled water); Group 3 was orally administered with diazinon (25 mg/kg body weight, dissolved in distilled water) (Ahmed et al., 2013 ▶) and, one hour later, silymarin (50 mg/kg body weight) (Kose et al., 2012 ▶). Groups 4, 5 and 6 were orally administered with diazinon (25 mg/kg body weight, dissolved in distilled water) (Ahmed et al., 2013 ▶) and, one hour later, they received *T. chebula* fruit extract (200, 400, and 800 mg/kg, respectively) (Bag et al., 2013 ▶; Sireeratawong et al., 2012 ▶). 

Fifteen days later, fasted rats were anesthetized with chloroform, their blood specimens were taken using cardiac puncture method and serum and plasma were separated. All serum and plasma specimens were stored at -80 °C until further analysis. In addition, a piece of the liver was removed to determine liver CAT, SOD, vitamin C, *TNF-α* gene expression, and to conduct histopathological studies. All procedures were approved by the Ethics Committee of Shahrekord University of Medical Sciences, Shahrekord, Iran.

**Biochemical analysis**

Levels of HDL-C, LDL-C, TG, TC, urea, creatinine, GPT, and GOT in the serum were measured according to enzymatic method using an atuoanalyzer (BT 3000, France). Serum TNF-α level was measured by an ELISA kit (BT-Laboratory, China). Very low density lipoprotein cholesterol (VLDL-C) was estimated according to method of Friedewald (1972). ▶

**Determining serum and tissue MDA levels**

Serum and tissue MDA levels were measured as described previously (Valipour et al., 2016 ▶). MDA levels were assessed using high-performance liquid chromatography (Agilent, USA) method with thiobarbituric acid as the reagent. The measurements were done in triplicates and the results were expressed in μM. MDA standards were prepared from 1, 1, 3, 3-tetraethoxypropane.

**Determination of ferric reducing/antioxidant power (FRAP)**

Plasma antioxidant capacity was measured using tripyridyl triazine as described previously (Heidarian and Soofiniya, 2011 ▶). In this procedure, the complex between Fe^2+^ and TPTZ gives a blue color with an absorbance read at 593 nm. FeSO_4_.7H_2_O was used as a standard of FRAP assay at a concentration range of 100-1000 μM.

**Determination of hepatic CAT and SOD activities**

Hepatic CAT activity was determined according to a previously described method (Heidarian et al., 2014b ▶). Hepatic SOD activity was measured by inhibition of NBT reduction at 560 nm using Beauchamp and Fridivich method (1971). Total protein was determined by Bradford (1976) ▶ method in homogenates.

**Determining liver vitamin C level**

Hepatic tissue vitamin C level was measured using 2,4 dinitrophenyl hydrazine (2,4 DNPH) according to the method of Omaye et al. (1979) ▶. Briefly, 100 mg of hepatic tissue was homogenized in 900 μl trichloroacetic acid 5% and centrifuged at 3500 g for 20 min. Then, 500 μl supernatant was dissolved in 100 μl 2,4 dinitrophenyl hydrazine/thiourea/copper (DTC) and incubated at 37°C for 3 hr. Next, 750 μl cold sulfuric acid was added to the reaction mixture and incubated at room temperature for 30 min. Then, the absorbance was read at 520 nm. A standard curve of vitamin C was plotted for a concentration range of 0-20 μg/μl. 

**Measuring serum protein carbonyl (PC)**

Serum PC was measured according to the method of Reznick and Packer (1994) ▶ using 6 M guanidine hydrochloride. The amount of carbonyl was expressed in nmol DNPH/mg protein. 

**Real-time quantitative PCR (RT-qPCR) for TNF-α**

Total mRNA (from samples of 1000 mg of each rat's liver) was extracted using Thermo scientific kit according to the manufacturer's instruction. The quality and quantity of total RNA were determined at 260/280 nm using Nanodrop 2000 Spectrophotometer (Thermo, USA) (Sahu et al., 2014 ▶). cDNA was amplified by RT-qPCR using SYBR® Green PCR Master Mix in the presence of specific primers for *TNF-α* (forward: 5'-CTGGCGTGTTCATCCGTTC-3', reverse: 5'-GGCTCTGAGGAGTAGACGATAA-3') and *β-actin* (forward: 5'-CGGTCAGGTCATCACTATCGG-3', reverse: 5'-TCTTTACGGATGTCAACGTCACAC-3') genes. The primers were designed using Oligo 6.0 software (Molecular Biology Insights, Cascade, Co.) and confirmed by blast (NCBI). The primers were purchased from Eurogentec (Seraing, Belgium). The proliferation steps were as follow: a first denaturation at 95°C for 10 min; RT-qPCR of 40 cycles with a three-step program (15 sec at 95°C for denaturation, 20 sec at 60°C for annealing, and 25 sec at 72°C for extension). *β-actin *was used as the internal control gene to normalize the gene expression data.

**Histopathological** **studies**

After the rats were sacrificed, their livers were taken out and fixed in formaldehyde 20% solution. After paraffin embedding, 5-μm thick sections were prepared and stained with hematoxylin and eosin (H and E) (Carleton et al., 1980 ▶) for photomicroscopic observation, especially infiltration of the inflammatory cells.

**Statistical analysis**

The data were expressed as mean ± SD and analyzed by one-way analysis of variance (ANOVA) using SPSS 20.0 (Chicago, IL). A *P *value <0.05 was considered statistically significant. Group means were compared using Tukey's *post-hoc* test for multiple comparisons.

## Results

Total phenolic, flavonoid, and flavonolic contents in the hydroalcoholic extract of *T*. *chebula *fruits were 326.83 ng/g, 32.15 ng/g, and 15.36 ng/g, respectively. The antioxidant activity of *T. Chebula* extract was 3.36 μg/ml. 

**Effect of** ***T. Chebula*** **fruits extract on serum parameters**

**Figure 1:**
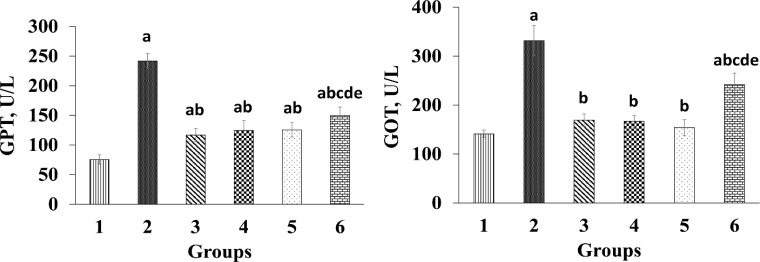
Effect of *Terminalia chebula *extract on GOT and GPT activities. Group 1 was the negative control; group 2 was diazinon-administered; group 3 was treated with diazinon and silymarin-administered, and groups 4-6 were administered with diazinon and *Terminalia chebula* hydroalcoholic fruits extract at the doses of 200, 400, and 800 mg/kg, respectively. ^a^ p<0.05 compared to group 1. ^b^ p<0.05 compared to group 2. ^c^ p<0.05 compared to group 3. ^d^ p<0.05 compared to group 4. ^e^ p<0.05 compared to group 5

The administration of diazinon caused a significant increase (p<0.05) in serum GOT and GPT levels in diazinon-administered rats compared to the control group (Figure. 1). Treatment with different doses of *T*. *chebula* significantly decreased (p<0.05) serum GOT and GPT levels compared to diazinon-administered rats (group 2). In silymarin-treated group a noticeable reduction (p<0.05) was observed in serum GOT and GPT compared to group 2 (diazinon-administered rats).

**Table 1:** Biochemical parameters in different groups of rats

**Parameters, mg/dl**	**Group 1**	**Group 2**	**Group 3**	**Group 4**	**Group 5**	**Group 6**
**TG**	65.12±7.71	153.37±13.55^a^	86.75±12.72^a^^b^	76.75±8.08^b^	91.87±13.21^a^^b^	147.7±9.11^a^^c^^d^^e^
**TC**	71.75±7.97	149.37±15.68^a^	81.12±6.83^b^	82.00±6.92^b^	89.62±8.78^a^^b^	144.5±10.75^a^^c^^d^^e^
**HDL**	49.41±2.51	25.5±2.64^a^	39.39±2.11^a^^b^	38.79±3.89^a^^b^	47.82±3.39^b^^c^^d^	26.13±3.23^a^^c^^d^^e^
**LDL**	12±2.07	10.00±3.29	11.50±1.61	11.62±1.18	13.77±1.09^b^	14.62±0.95^b^^c^^d^
**VLDL**	13.41±1.27	30.72±2.71^a^	17.50±2.35^a^^b^	15.35±1.61^b^	18.02±2.71^a^^b^	29.1±2.13^a^^c^^d^^e^
**Urea**	46.87±2.47	36.87±2.92^a^	48.37±3.66^b^	47.37±5.15^b^	51.00±4.95^b^	47.37±3.15^b^
**Creatinine**	0.43±0.05	0.43±0.07	0.45±0.05	0.43±0.05	0.51±0.09	0.57±0.07^a^^b^^c^^d^

Group 1 was the negative control; group 2 was diazinon-administered; group 3 was treated with diazinon and silymarin, and groups 4-6 were administered with diazinon and *Terminalia chebula *hydroalcoholic fruits extract at the doses of 200, 400, and 800 mg/kg, respectively.

a p<0.05 compared to group 1.

b p<0.05 compared to group 2.

c p<0.05 compared to group 3.

d p<0.05 compared to group 4.

e p<0.05 compared to group 5.

Table 1 shows the effects of *T*. *chebula *fruits extract on serum lipids and certain biochemical parameters in the experimental groups. Diazinon resulted in a significant increase (p<0.05) in serum TG, TC, and VLDL levels in the rats administered with diazinon-only compared to the control group. A significant decline (p<0.05) was observed in serum TG, TC, and VLDL levels between rats treated with 200 and 400 mg/kg of *T. chebula* fruits extract compared to the rats administered with diazinon-only (Table 1). Moreover, serum TG, TC, and VLDL levels were not significantly different between the group administered with 800 mg/kg *T*. *chebula *fruits extract and diazinon-only treated group (group 2). Serum HDL level significantly decreased (p<0.05) in diazinon-administered rats compared to the control group. In groups 4 and 5 (treated with 200 and 400 mg/kg* T. chebula *fruits extract, respectively), serum HDL level increased significantly (p<0.05) compared to the diazinon-only treated group (group 2). 

Administration with diazinon did not significantly change (p>0.05) in creatinine level in diazinon-administered group compared to the control group (Table 1). However, in the rats administered with 800 mg/kg *T*. *chebula* fruits extract, a significant increase (p<0.05) was seen in serum creatinine level compared to other groups except for the group 4 (administered with 400 mg/kg *T. chebula* extract). Administration with diazinon caused a significant decrease (p<0.05) in serum urea level in the diazinon-administered rats compared to the control group (Table 1). Nevertheless, administration of *T*. *chebula *fruits extract caused a significant increase (p<0.05) in the serum urea level compared to the rats administered with diazinon-only.

**The effect of** ***T. Chebula*** **fruits extract on liver CAT, SOD, and vitamin C**

**Figure 2.**
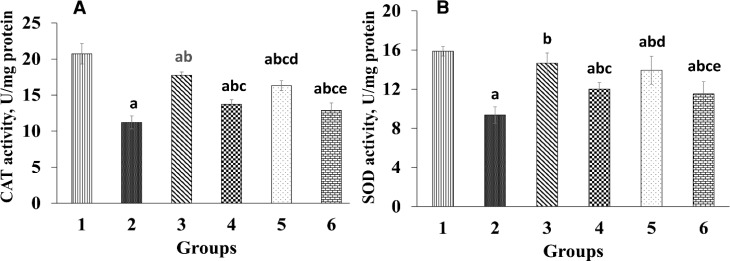
Effect *Terminalia chebula *extract on catalase (CAT) and superoxide dismutase (SOD) activities. Group 1 was the negative control; group 2 was diazinon-administered; group 3 was treated with diazinon and silymarin-administered, and groups 4-6 were administered with diazinon and *Terminalia chebula* hydroalcoholic fruits extract at the doses of 200, 400, and 800 mg/kg, respectively. ^a^ p<0.05 compared to group 1. ^b^ p<0.05 compared to group 2. ^c^ p<0.05 compared to group 3. ^d^ p<0.05 compared to group 4. ^e^ p<0.05 compared to group 5

**Figure 3:**
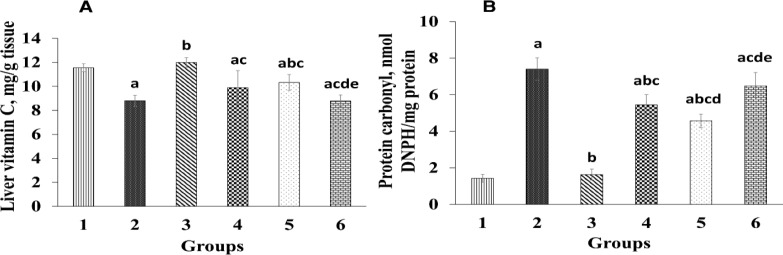
Effect of *Terminalia chebula *extract on liver vitamin C and serum protein carbonyl. Group 1 was the negative control; group 2 was diazinon-administered; group 3 was treated with diazinon and silymarin-administered, and groups 4-6 were administered with diazinon and *Terminalia chebula* hydroalcoholic fruits extract at the doses of 200, 400, and 800 mg/kg, respectively. ^a^ p<0.05 compared to group 1. ^b^ p<0.05 compared to group 2. ^c^ p<0.05 compared to group 3. ^d^ p<0.05 compared to group 4. ^e^ p<0.05 compared to group 5

In rats administered with diazinon-only (group 2), a significant decrease (p<0.05) in liver vitamin C, CAT, and SOD levels was seen compared to the control group (Figures 2 and 3A). Hepatic CAT and SOD activities significantly increased (p<0.05) in rats that received *T*. *chebula *fruits extract compared to those administrated with diazinon-only (group 2). The rats administered with 400 mg/kg *T*. *chebula *fruits extract exhibited a significant increase (p<0.05) in vitamin C level in contrast to the rats administrated with diazinon-only (Figure 3A). In silymarin-treated group, a noticeable elevation (p<0.05) was observed in liver vitamin C level compared to diazinon-only treated group (group 2).

**The effect of** ***T. chebula*** **fruits extract on serum protein carbonyl (PC)**

Figure 3B shows the effect of *T. chebula* fruits extract on PC in experimental groups. Serum PC content increased significantly (p<0.05) in rats administered with diazinon compared to the control group. However, serum PC level in rats administrated with diazinon plus 200 and 400 mg/kg *T*. *chebula *fruits extract significantly decreased (p<0.05) compared to the rats administrated with diazinon-only (group 2). 

. 

**The effect of** ***T. chebula*** **fruits extract on liver and serum MDA and FRAP levels**

Table 2 shows the effects of* T*. *chebula *fruits extract on plasma FRAP, serum and liver MDA levels in the experimental groups. Administration of diazinon to group 2 (diazinon-only treated group) caused a significant decrease (p<0.05) in plasma FRAP compared to the control group. In groups 4 and 5 (rats received 200 and 400 mg/kg *T*. *chebula *fruits extract, respectively), however, a noticeable increase was seen (p<0.05) in plasma FRAP compared to diazinon-only treated group (group 2). Also, in group 3 (treated with 50 mg/kg silymarin), a signiﬁcant elevation (p<0.05) was observed in plasma FRAP as compared to diazinon-only treated group. 

Administration of diazinon to group 2 (diazinon-only terated group) led to a significant elevation (p<0.05) of the serum and liver tissue MDA levels compared to the control (Table 2). In groups treated with *T*. *chebula *fruits extract, serum and liver tissue MDA significantly decreased (p<0.05) compared to group 2 (diazinon-only treated group). Moreover, liver MDA levels in group 3 (50 mg/kg silymarin) showed a noticeable decline (p<0.05) compared to the groups treated with all doses of *T*. *chebula *fruits extract (Table 2). 

**The effect of** ***T. chebula*** **fruits extract on diazinon-induced inflammation of the liver**

**Figure 4:**
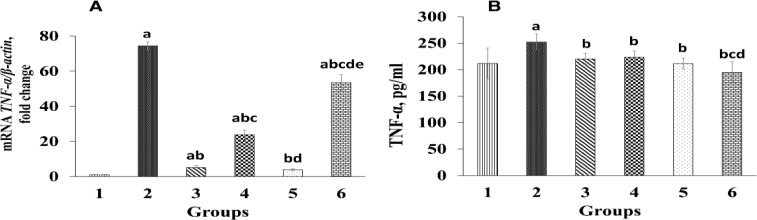
Effect of *Terminalia chebula *extract on serum level of tumor necrosis factor-α (TNF-α) and the relative expression of *TNF-α* in the liver. Group 1 was the negative control; group 2 was diazinon-administered; group 3 was treated with diazinon and silymarin-administered, and groups 4-6 were administered with diazinon and *Terminalia chebula* hydroalcoholic fruits extract at the doses of 200, 400, and 800 mg/kg, respectively.

^a ^p<0.05 compared to group 1. ^b ^p<0.05 compared to group 2. ^c ^p<0.05 compared to group 3. ^d ^p<0.05 compared to group 4. ^e ^p<0.05 compared to group 5.

Diazinon administration to group 2 caused a significant increase (p<0.05) in the expression of *TNF-α* gene compared to the control group (Figure 4A). Administration of *T*. *chebula* fruits extract led to a significant reduction (p*<*0.05) in *TNF-α* gene expression compared to group 2 (diazinon-only treated group). In addition, a signiﬁcant reduction (p<0.05) was observed in *TNF-α* gene expression between group 3 (50 mg/kg silymarin) and group 2 (diazinon-only treated group). 

Figure 4B shows the effect of *T*. *chebula *fruits extract on serum TNF-α level. Serum TNF-α level was significantly higher (p<0.05) in the rats administered with diazinon than the control group. Oral administration of *T*. *chebula *fruits extract, however, significantly decreased (p<0.05) serum TNF-α level compared to group 2 (diazinon-only treated group) in a dose-dependent manner. 

**Table 2:** Serum and tissue liver malondialdehyde and plasma FRAP levels.

**Parameters, μM **	**Group 1**	**Group 2**	**Group 3**	**Group 4**	**Group 5**	**Group 6**
**Serum MDA **	1.73±0.35	5.51±0.82^a^	1.43±0.36^b^	3.52±0.37^a^^b^^c^	2.67±0.36^a^^b^^c^^d^	2.85±0.39^a^^b^^c^
**Liver MDA**	2.38±0.35	8.72±0.82^a^	2.42±0.45^b^	6.37±0.72^a^^b^^c^	4.42±0.63^a^^b^^c^^d^	3.52±0.40^a^^b^^c^^d^^e^
**plasma FRAP**	358.45±17.79	234.32±25.38^a^	365.07±18.41^b^	308.2±20.92^a^^b^^c^	347.32±27.07^b^^d^	237.07±21.7^a^^c^^d^^e^

Group 1 was the negative control; group 2 was diazinon-administered; group 3 was treated with diazinon and silymarin-administered, and groups 4-6 were administered with diazinon and *Terminalia chebula* hydroalcoholic fruits extract at the doses of 200, 400, and 800 mg/kg, respectively.

a p<0.05 compared to group 1.

b p<0.05 compared to group 2.

c p<0.05 compared to group 3.

d p<0.05 compared to group 4.

e p<0.05 compared to group 5.

**Histopathological examinations**

**Figure 5:**
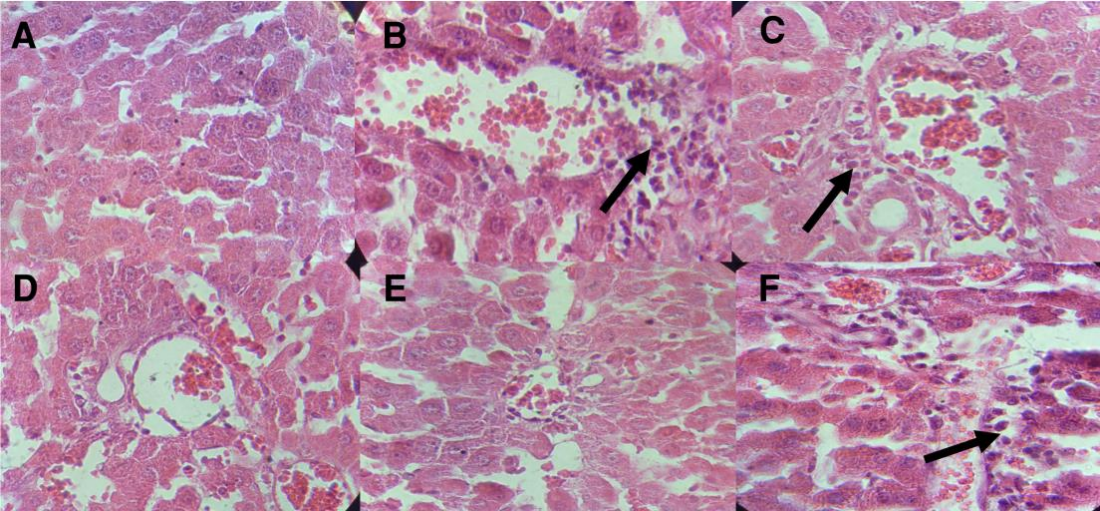
Effect of *Terminalia chebula *extract on pathological changes in the liver of different groups. **A**, the liver section of the control rats (group 1) showing normal structure. **B**, diazinon-administered rats (group 2) showing pathological changes in the liver such as mononuclear cell infiltrations (black arrow). **C**, diazinon-administered rats treated with silymarin (group 3). **D**, **E**, and **F **(groups 4 to 6) diazinon-administered rats treated with 200, 400, and 800 mg/kg body weight of *T. chebula* fruits extract, respectively. Treatment with *T. chebula *fruit extract led to a reduction in inflammatory cells when compared with diazinon-only treated rats. The arrows show inflammatory cells

Figure 5 shows *T*. *chebula* fruits extract effects on histopathological changes in all experimental groups. Oral administration of diazinon to gourp 2 (diazinon-only treated group) led to lymphocyte cell infiltration compared to the control group (Figures 5A and B). Liver tissue in the group administered with 200 mg/kg *T*. *chebula *fruits extract resulted in a reduction in inflammatory cell infiltration compared to diazinon-only treated group (Figure 5D). Liver lymphocyte cell infiltration decreased in group 5 (treated with diazinon and 400 mg/kg *T*. *chebula *fruits extract) compared to group 2 (diazinon-only treated group) (Figure 5E). Microscopic examinations of liver tissues from group 6 (treated with diazinon and 800 mg/kg *T*. *chebula *fruits extract) demonstrated an increase in lymphocytes infiltration (Figure 5F).

## Discussion

Exposure to diazinon, a synthetic insecticide, causes damages to hepatocytes (Gokcimen et al., 2007 ▶) due to production of ROS and weakened antioxidant system (Lukaszewicz-Hussain, 2010 ▶). Serum GOT and GPT levels are considered as indices of liver injury and increased levels of these enzymes indicate liver injury (Gokcimen et al., 2007 ▶). In this study, diazinon administration caused liver dysfunction and significant increases in serum GOT and GPT levels compared to the control group (Figure. 1), which is in agreement with a previous study (Gokcimen et al., 2007 ▶). However, *T. chebula* caused significant decrease in serum GOT and GPT levels due to its antioxidant properties. Previous studies have demonstrated that antioxidants can protect cell membrane integrity and decrease enzyme leakage through free radical scavenging activities (Karimi-Khouzani et al., 2017 ▶; Messarah et al., 2013 ▶). *T. chebula* extract contains large amounts of tannins, flavonoids, sterols, resins, fructose, aminoacids, and fixed oils (Chandra, 2012 ▶) .Therefore, the presence of flavonoids and other compounds in *T. chebula *extract may be, at least in part, responsible for its protective effects on diazinon-induced liver injury in this study.

In the present study, 15-day administration of diazinon led to a significant decrease in serum urea level compared to control group. The defective liver function caused decrease in serum urea (Lichter-Konecki, 2016 ▶) because urea is made by liver. However, in this study treatment with *T. chebula* reduced the liver injury (Figure 5) and restored the metabolism of urea in the liver due to, at least in part, its antioxidant properties.

Organophosphates are metabolized by cytochrome P450 and oxidative stress can cause lipid peroxidation which results in increased levels of PC and MDA (Lukaszewicz-Hussain, 2010 ▶). In the present study, serum and tissue MDA and PC levels increased significantly in group 2 (diazinon-only treated group) compared to the control group (Table 2 and Figure 3B), which is consistent with previous studies (El-Shenawy et al., 2010 ▶; **Razavi et al., 2013**). In our study, *T. chebula* at 200 and 400 mg/kg doses along with silymarin significantly decreased PC and MDA levels as compared to group 2 (diazinon-only treated group). A study demonstrated the protective effects of saffron in reduction of MDA levels in diazinon-induced hepatotoxicity (Lari et al., 2015 ▶). Also, previous works have shown that *T. chebula* has considerable antioxidant properties (Chandra, 2012 ▶). In this study, *T. chebula* not only reduces serum MDA, as a lipid-peroxidation index, but also elevated plasma FRAP concentrations (Table 2). Therefore, an increase in FRAP, due to antioxidant properties of *T.* *chebula* extract, at least in part, can explain the decrease in liver MDA (which was dose-dependently) and PC in the group administered with this extract compared to group 2 (diazinon-only treated group).

Additionally, dyslipidemia is seen in many diseases (Heidarian and Rafieian-Kopaei, 2013 ▶). In the present study, diazinon administration caused an increase in serum TG, TC, and VLDL levels and a decrease in serum HDL level in group 2 (diazinon-only treated group) compared to control group which is in agreement with previous studies (Heidarian and Rafieian-Kopaei, 2013 ▶; Omar et al., 2016 ▶). Nevertheless, serum LDL level did not change significantly, which is agreement with previous studies (Al-Attar and Abu Zeid, 2013 ▶; Heidarian and Rafieian-Kopaei, 2013 ▶). In this study, treatment with *T. chebula* extract (200 and 400 mg/kg) or silymarin decreased serum VLDL, TG, and TC levels compared to group 2 (diazinon-only treated group) (Table 1), whereas *T. chebula* led to a considerable increase in HDL levels in treated groups. On the other hand, in our study *T. chebula* extract at 800 mg/kg dose did not have significant beneficial effects on diazinon-induced liver toxicity and oxidative stress in rats. A previous study showed that the ethanolic extract of *T. chebula* fruits at doses less than 500 mg/kg body weight, demonstrated no toxic effects (Kannan et al., 2012 ▶). Therefore, in our study elevation in the levels of GOT, GPT, PC, TG, TC, VLDL-C, and Cr at 800 mg/kg of *T. chebula* fruits extract may be, at least in part, due to the side effects of *T. chebula* extract at higher doses. It is reported that, herbal remedies can cause harm, especially to the liver, at higher doses through a wide range of mechanisms (Posadzki et al., 2013 ▶). On the other hand, several reports have demonstrated that natural agents can reduce hyperlipidemia, which is in agreement with our results (Heidarian et al., 2014a ▶; Korou et al., 2016 ▶; Nouri et al., 2017 ▶). It has been reported that silymarin is a lipid-lowering therapeutic agent which leads to adjustment of serum dyslipidemia (Heidarian and Rafieian-Kopaei, 2012 ▶). In this study, however,* T. chebula* extract at the dose of 400 mg/kg had, at least in part, a remarkable effect on the reduction of serum lipid levels compared to silymarin due to its antioxidant properties. 

Exposure to insecticides induces the production of free radicals by inducing oxidative stress, which results in an imbalance in antioxidant system (Al-Attar and Abu Zeid, 2013 ▶). In this study administration of diazinon weakened enzymatic (SOD and CAT) and non-enzymatic (vitamin C) antioxidant defense system, which is consistent with previous studies (Abdel-Daim, 2016 ▶; Balkan et al., 2002 ▶). However, administration of *T. chebula *fruits extract led to an elevation in vitamin C, SOD, and CAT levels (Figures 2 and 3A) in treated groups compared to group 2 (diazinon-only treated group). In addition, previous studies have shown that diazinon causes histopathological changes in liver tissue and results in widespread necrosis (Gokcimen et al., 2007 ▶). Thus, in this study the elevations in vitamin C, SOD, and CAT levels can be, at least in part, considered as an additional reason for reducing ROS, serum and liver MDA, protein carbonyl, and liver structural and pathological abnormalities in *T. chebula *extract treated groups which, in turn, leads to improvement of liver histopathological results (Figure 5). 

TNF-α, as an inflammatory cytokine, is one of the most important indices of oxidative stress-induced inflammation that can affect proliferation and induce apoptosis (Hariri et al., 2010 ▶; Wullaert et al., 2006 ▶). Several studies have demonstrated that diazinon-induced hepatotoxicity is structurally associated with inflammation and is considerably associated with an increase in TNF-α (Hariri et al., 2010 ▶) which was also observed in our study (Figure 4). The present study demonstrated that hepatic *TNF-α* expression and serum TNF-α level increased significantly in group 2 (diazinon-only treated group) compared to the control. In this study, treatment with the extract of *T. chebula *fruits or silymarin significantly decreased serum TNF-α and its gene expression in liver tissues (Figure 4) which is in accordance with earlier studies (Ansari et al., 2016 ▶; Valipour et al., 2016 ▶). Furthermore, administration of crocin and safranal to diazinon-treated animals, has been demonstrated to lower TNF-α level compared to animals treated with diazinon only (Hariri et al., 2010 ▶). Thus, the reduction in serum TNF-α and *TNF-α* expression in liver tissues, is another evidence, at least in part, which confirms liver protective effects of *T. chebula *fruits extract due to its antioxidant activities. Nevertheless, TNF-α is a non-specific liver marker which is expressed in different cells and organs (such as monocytes, kidney, and the brain) as an inflammatory cytokine (Donnahoo et al., 1999 ▶). Therefore, slight reductions in serum TNF-α in groups treated with 200 and 400 mg/kg of *T. chebula* fruits extract as compared to those of group 2, in spite of the considerable reduction of *TNF-α* expression in liver tissues (Figure 4), can be resulted from releases from other tissues.

Diazinon may induce apoptosis through activating caspase 9 and 3 and increasing Bax/Bcl2 (Lari et al., 2015 ▶). In this study, we did not evaluate the effect of *T. chebula *fruits extract on liver apoptosis/necrosis, pro-apoptotic factors such as NF-κB and p53 or down-regulation of the activities of some anti-apoptotic proteins such as Bcl-2. These factors can influence cell apoptosis and survival. Therefore, future studies should focus on the anti-apoptotic effects of *T. chebula *fruits extract.

The results of this study demonstrated that *T. chebula* fruits extract protected liver against diazinon-induced hepatotoxicity in male rats. The protective effects of *T. chebula* fruits extract can be related to its antioxidant and anti-inflammatory properties. Therefore, *T. chebula* fruits extract can be considered as a protective agent against free radical-induced liver damage caused following exposure to diazinon and this extract can minimize the toxic effects of diazinon in rats.
